# Effect of a solar-powered spatial emanator on malaria prevalence in Uganda (Mossie-GO trial): A double-blinded, cluster-randomised, and placebo-controlled trial

**DOI:** 10.1371/journal.pgph.0007457

**Published:** 2026-11-05

**Authors:** Jessica Dennehy, Will Dyall, Akin Jenkins, Asadu Sserwanga, Ruth Kigozi, Justine Babirye, John Baptiste Bwanika, Megan Arnett, James Oduor, Anthony Nuwa, Peter Ojangole, Henry D. Mawejje, Matthew A. Turner, Richard Wallace, Frederick G. A. Lyle, Jeff Hebden, John Bradley, Alexandra Hiscox, James G. Logan, Jane Achan, Alastair K. Livesey, Robert T. Jones

**Affiliations:** 1 Digital Odour Technologies Ltd, Dagenham, United Kingdom; 2 Malaria Consortium, Kampala, Uganda; 3 Department of Disease Control, London School of Hygiene & Tropical Medicine, London, United Kingdom; 4 Kua Solar Ltd, London, United Kingdom; 5 Department of Chemistry, Loughborough University, Loughborough, Leicestershire, United Kingdom; 6 Africa Power Ltd, Cowfold, United Kingdom; 7 Department of Infectious Disease Epidemiology, London School of Hygiene & Tropical Medicine, London, United Kingdom; Tulane University School of Public Health and Tropical Medicine, UNITED STATES OF AMERICA

## Abstract

The continued threat of malaria has driven investigations into the efficacy of a range of new tools, including spatial emanators which release active ingredients that can affect mosquito host-seeking vector behaviour and reduce human contact. This study aimed to estimate the effect of a novel solar-powered spatial emanator in reducing the prevalence of malaria in rural and peri-urban settings. The Mossie-GO trial was a prospective, cluster-randomised, controlled trial conducted in Jinja and Buikwe in Eastern Uganda. Fifty-six clusters were randomised at a 1:1 ratio to receive either the spatial emanator with transfluthrin-treated ‘coins’ or an identical device with no-active ingredient. Up to 100 houses per cluster were enrolled. Investigators, field staff, and study participants were masked to cluster allocation. The primary outcome was malaria infection prevalence among children aged ≤5 years at 12 months, compared between intervention and control clusters and adjusted for baseline cluster-level prevalence. The trial was registered with ClinicalTrials.gov (NCT06232954). The baseline-adjusted analysis did not show evidence of lower malaria infection prevalence in intervention clusters compared with control clusters at follow-up. The unadjusted OR for malaria infection was 0.63 (95% CI: 0.29–1.36, p = 0.239), and the baseline-adjusted OR was 0.96 (95% CI: 0.66–1.39, p = 0.813). No significant differences in mosquito density were observed. No serious adverse events were reported and there was no difference in the rate of adverse events between trial arms. The trial did not demonstrate significantly lower follow-up malaria infection prevalence in intervention clusters after adjustment for baseline cluster-level prevalence. Exploratory subgroup analyses reveal some indication of change and should be interpreted as hypothesis-generating. There was also an observed but not significant reduction in human biting rate in intervention households. Large variation in baseline data and between clusters may have limited the power of the study to detect significant results.

## Introduction

It is estimated that 1.7 billion malaria cases and 12 million deaths have been averted in the World Health Organization’s Africa region since 2000 [1]. This has primarily been achieved through unprecedented levels of intervention coverage across sub-Saharan Africa, including insecticide treated nets (ITNs), indoor residual spraying, and artemisinin-based combination therapies [2], but also likely reflects other factors that modify malaria transmission, such as improvements in socioeconomic conditions, infrastructure, and housing [3].

Despite this progress, Africa continues to carry the heaviest burden of malaria mortality, with approximately 95% of all malaria deaths worldwide, and momentum towards achieving global reduction targets has stalled [1]. Innovative solutions are required, particularly given the challenges from insecticide resistance and changing vector behaviours. Recently-trialled approaches have included the mass distribution of ivermectin as an adjunct to dihydroartemisinin–piperaquine [4], delivery of attractive targeted sugar baits [5], and installation of insecticide-based durable wall linings [6]. Ochomo et al [7] investigated the protective efficacy of a spatial emanator. Their study involved the distribution of a transfluthrin-based passive emanator in districts in western Kenya, close to the border with Uganda, and evaluated the reduction in risk of malaria infection in a setting characterised by high malaria transmission, pyrethroid resistant malaria vectors, and high coverage of long-lasting insecticidal nets. In addition to other studies, it contributed to the recent WHO recommendation supporting the use of spatial emanators, introducing a new class of intervention for malaria control and prequalification of two spatial emanator products [8].

Spatial emanators present an exciting means of mosquito bite prevention by deterring host blood-feeding [9]. The volatile pyrethroid transfluthrin has been investigated as a spatial repellent in a number of different formats to protect indoor and outdoor spaces, including in eave strips [10], sandals [11], chairs [12], coils [13], decorative sisal baskets [14], and the pre-treated plastic film passive emanators used in the study by Ochomo and colleagues [7]. In addition to having spatial repellent properties, transfluthrin has been shown to inhibit feeding, decrease fecundity, and cause mortality in mosquitoes [15–17]. These additional effects mean that transfluthrin-based tools could contribute to community-wide protection, even for those individuals or households that are non-users [17].

Release rates are central to the efficacy of spatial emanators. Spatial repellent compounds can evaporate at ambient temperature without the need for an external source of energy, but lower rates of protection have been reported in some studies where cooler conditions lowered evaporation rates of the active ingredient [18,19]. To overcome the limitations of passive devices, commercial products typically rely on an external power source or combustion. However, low-cost coils and kerosene oil lamps that release transfluthrin produce smoke, which may have harmful effects inside houses, and electrical emanators are typically not affordable in low-income settings [13,20,21]. A low-energy, solar-powered device can provide controlled and continuous emanation of transfluthrin and offer a cost-effective option in off-grid settings. This could be particularly valuable at those times when vectors are active but people are not yet sleeping under a bed net [22].

This trial was designed to estimate the effect of Mossie-GO, a solar-powered transfluthrin-based active spatial emanator, on malaria infection prevalence among children aged ≤5 years, using repeated cross-sectional surveys, as determined by rapid diagnostic test (RDT) and microscopy. Additionally, the trial sought to measure the impact of the intervention on entomological correlates of transmission, user acceptance of the device, quantification of transfluthrin concentration in the air, and the safety of the intervention.

## Methods

### Ethics statement

The protocol and associated study materials were approved by the Vector Control Division - Research Ethics Committee in Uganda (VCDR-2023-40) and the Uganda National Council for Science and Technology (UNCST. HS419ES), with additional approval for focus group studies by the LSHTM MSc Ethics Committee (reference: 31773/RR/38386). Written informed consent was gained for all participation in the study. Written informed consent was obtained from the parent/guardian of each participant under 18 years of age.

### Study design

The Mossie-GO trial was a cluster-randomised, placebo-controlled trial blinded to both participants and investigators. The trial was registered with ClinicalTrials.gov on 04/01/2024 (NCT06232954; https://clinicaltrials.gov/study/NCT06232954). The protocol has been published [23] (see Supporting information for protocol and access to statistical analysis plan). These contain full details of sample size calculations, interim analyses, randomization, blinding and statistical analyses. This trial has been reported according to CONSORT guidance [24].

### Participants

The trial was conducted in the districts of Jinja and Buikwe, Eastern Uganda, with moderate to high malaria burden and stable perennial malaria transmission. In these districts, 478 clusters were assessed for eligibility, of which 82 met the inclusion criterion of more than 150 households with a child aged ≤5 years. From these, 56 clusters were selected at random for inclusion in the trial. Each cluster was a distinct village separated from another cluster by at least 365 m. All clusters received national distribution of ITNs during the period 2022 prior to study initiation.

A sensitisation campaign was delivered by Village Health Teams (VHTs) before the intervention. Written informed consent was gained for all participation in the study by VHTs. This included consent from heads of household for placement of the spatial emanator and for engagement in household and acceptability surveys. Additional consent was obtained from those heads of households where malaria, entomological or air sampling activities took place. Entomological sampling included indoor and outdoor human landing catches (HLCs) and mosquito collection using Centers for Disease Control (CDC) light traps. Consent for focus group discussions (FGDs) was also obtained separately.

At baseline, 9,450 devices were deployed to enrolled households. Within a cluster, all households with a child ≤ 5 years of age received a device, and remaining households were selected at random to receive the remaining devices. To be eligible for enrolment into the primary endpoint survey (malaria blood sampling), participants needed to meet the inclusion criteria of age (0 months to ≤ 5 years), no plans for extended travel (>1 month) outside of home during the study, not participating in another clinical trial or being on regular malaria prophylaxis, and parents or guardians providing written consent. Secondary endpoint surveys (entomological and air sampling) were nested within the primary endpoint sample population randomly selected at each time point (Fig 1). Recruitment for the intervention study began on 08/06/2024 and ended on 23/06/2024. Recruitment for focus groups began on 12/06/2025 and ended 26/06/2025.

**Fig 1:**
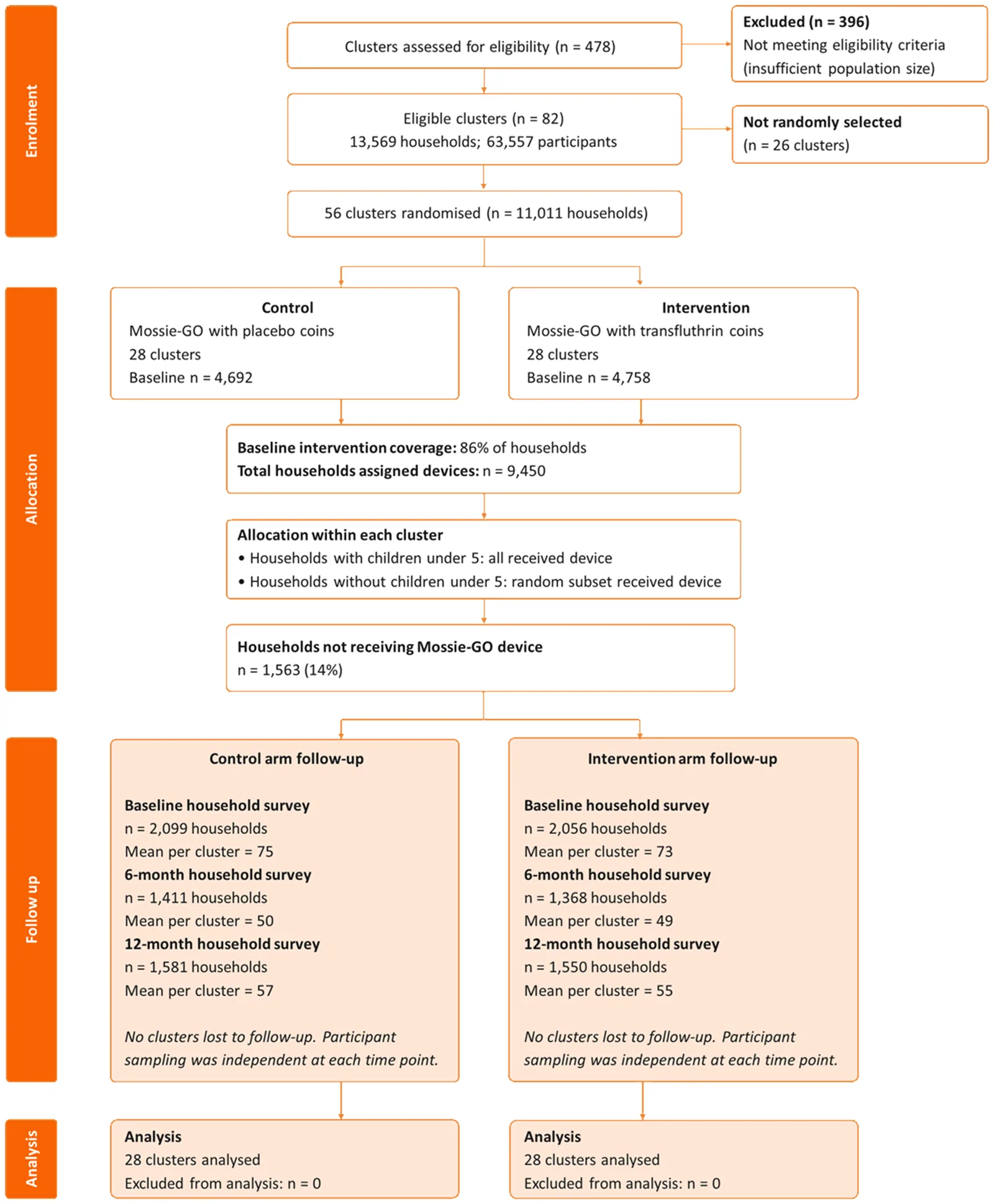
Trial flow chart. **Progress of clusters and participants through the phases of the trial: enrolment, randomisation, allocation, follow-up, and analysis.** Of 478 clusters assessed for eligibility, 82 met the inclusion criterion of more than 150 households with a child aged ≤ 5 years. Fifty-six clusters were randomly selected and randomised 1:1 to intervention or control arms. Household survey sampling was conducted independently at each time point. No clusters were lost after randomisation.

### Randomisation and masking

Cluster selection, randomisation and intervention allocation occurred in April-June 2024, prior to baseline sampling. An independent statistician assigned the 56 clusters to one of the two groups (intervention or control) in a 1:1 ratio using unrestricted randomisation. There was an average of 197 households per cluster with an average of 170 households per cluster enrolled into the study to receive the spatial emanator. All enrolled households received the intervention device or a control device containing no active ingredient. Funding and study timelines did not permit a baseline analysis to inform the removal of clusters, or to balance clusters between study arms, based on malaria prevalence; in a reported deviation from protocol, cluster selection did not include data on baseline malaria prevalence. For all study endpoints, excluding air sampling, clusters were allocated to a 1:1 intervention to control ratio (Table 1). Air sampling clusters were allocated to a 1:6 ratio to maximise available resources. Selection of clusters for air sampling was conducted by the delegated unblinded staff member.

**Table 1:** Number of total, intervention and control clusters and households per sampling endpoint.

Endpoint	Clusters			Households per cluster
	Total	Intervention	Control	
Primary				
Malaria sampling	56	28	28	60-100
Secondary				
Indoor light trapping	56	28	28	7
Human landing catches	10	5	5	3
Household and acceptability survey	56	28	28	60-100
Air sampling	7	6	1	2

The intervention was the Mossie-GO device, a solar-powered transfluthrin-based active emanator, manufactured by Africa Power Ltd. The spatial emanator was coupled with a solar panel, charging cable and either a transfluthrin-treated (intervention arm) or blank (control arm) refill cotton-based coin. The coin was replaced at monthly intervals by a member of the study team and used coins taken away for disposal. Both the intervention and control products were packaged identically (Fig 2).

**Fig 2:**
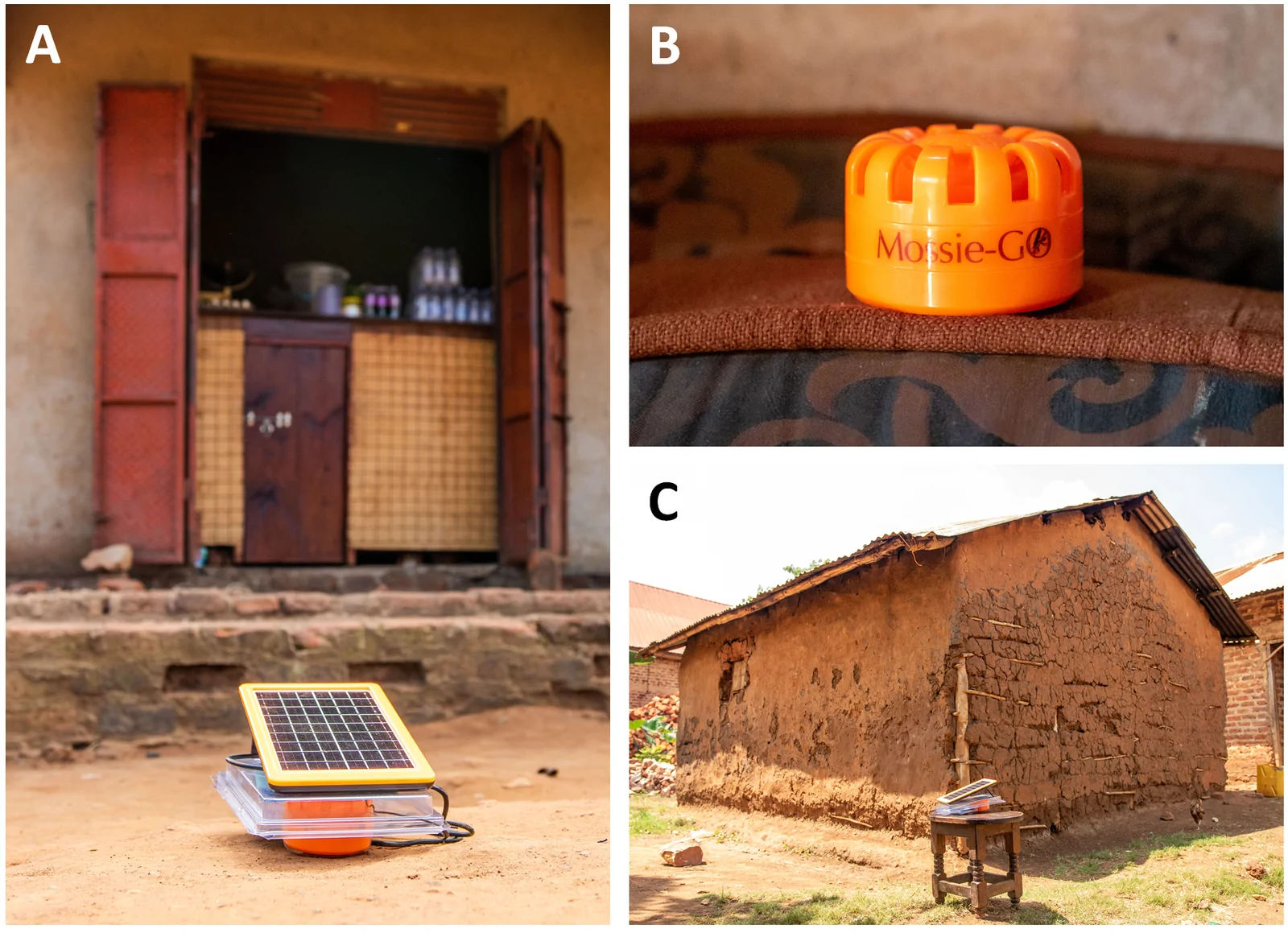
The spatial emanator being charged by its solar panel (A), the device in situ for overnight use (B), and a house with corrugated steel sheet roof and mud-brick walls, typical of houses in the rural clusters (C).

Only the independent statistician and two delegated staff members, responsible for coordinating the manufacture and distribution of the spatial emanators to either the intervention or control clusters, were unmasked. Before delivery to the study site, the independent device manufacturer applied labels to the spatial emanators and packs containing the intervention and control refill coins. There were no accidental instances of unmasking. The study did not withhold standard of care for clinical management of malaria or vector control interventions in either trial arm, however, use of these was monitored at each sampling time point.

### Procedures

Households were enrolled into the study and the intervention was disseminated at baseline (month 0). One hundred children per cluster at baseline, and sixty children per cluster at months 6 and 12, were selected at random from separate households for malaria infection testing. Participant selection was independent at each time point. A fingerstick blood sample was taken for a malaria rapid diagnostic test (RDT) and a blood smear for analysis by microscopy. All participants were eligible for malaria treatment if they test positive by RDT. Microscopic examination of slides was conducted twice by independent microscopists in reference laboratories. Primary endpoint sample sizes were selected to detect a between-arm difference in malaria infection prevalence, assuming a prevalence of malaria infection by RDT in children ≤ 5 years of age to be 20%, a coefficient of variation of 0.5 for between cluster prevalence, intervention effect of 40% and 90% power.

At all time points, a questionnaire was presented to the head of household at the point of malaria sampling to assess household characteristics and behaviours that may impact study outcomes. Questions within the survey included aspects of housing characteristics (room size, ventilation, construction materials), use of alternative vector control tools, number of inhabitants and their age, and presence of domesticated animals. In addition, at month 6 and 12, a questionnaire was presented to the head of household to assess whether households had positive or negative experiences with the spatial emanator and whether they would recommend it to other people, and determine their willingness to pay for the device. The surveys were conducted by the VHTs responsible for obtaining informed consent.

FGDs were conducted after the endline survey to further investigate acceptability of the intervention, including satisfaction with the device and participant recommendations for design modification. These sessions were also used to gauge participant knowledge of malaria and their approaches to malaria control. There were seven FGDs with end users, two with VHTs, and one with District Health Teams. Audio recordings of FGDs were manually transcribed verbatim and translated into English. Text was organised in NVivo (version 15. Lumivero, Denver, Colorado), with a combination of inductive and deductive coding for thematic analysis [25,26].

Within each of the 56 clusters, indoor light trap collections were conducted in seven households from 7 pm – 7 am for one night using miniature CDC light traps (John W. Hock Company, Gainesville, Florida). These collections were made at baseline, 6 and 12 months. Data collectors were trained in setting up and taking down light traps, and heads of households were trained to switch on/off the traps at the required time when necessary. Households were selected at random for CDC light trapping at each time point. Additionally, HLCs were performed indoors and outdoors at 6 pm to midnight for three consecutive nights in three households within each of ten clusters (30 households total) at baseline and at 6 months. Due to low catches, HLC at 12 months were extended to cover the period 6 pm to 6 am. This change was introduced to improve mosquito catches and is explicitly reported here to distinguish the 12-month HLC procedure from that used at earlier timepoints. Alongside the data collector, the head of household and community members were trained to conduct the HLC and were supervised during data collection. The clusters and households selected for HLCs and light trapping were selected at random for each time point.

Mosquitoes collected from light trapping and HLCs were morphologically identified, and all *Anopheles* specimens were preserved to record vector density and blood-fed status at each time point. Samples were all preserved. To characterize the insecticide susceptibility in the vector population in the study area, a series of insecticide assays were conducted. *Anopheles* larvae were collected by dipping at larval development sites identified across both trial arms. The larvae were reared to adults and tested for insecticide resistance following WHO tube test and CDC bottle assay protocols [27,28]. Permethrin was tested in tube tests at the diagnostic concentration of 0.75% and transfluthrin was tested in bottle assays at 2 µg/ml. Larval collections and insecticide testing took place in June and July 2025, corresponding with the end of the trial period.

Air sampling was conducted at 9 and 12 months in a total of 14 households, made up of two households from seven clusters, selected from the same pool of households undergoing HLCs. Two samples were collected at each household: in the evening approximately one hour after the spatial emanator had been switched on and then automatically four hours later, to determine variation in transfluthrin in the air. Sampling took place at a distance between 1–4 m from the spatial emanator and 0.5 m above the ground.

The air sampling was conducted using portable air sampling machines.An initial round of air sampling was conducted at baseline test in a hotel room using a Markes International ACTI-VOC PLUS pump kit. For sampling in participant houses, custom-built air samplers were used. These were designed to quantify airborne concentrations of particles within domestic indoor environments. The machines were powered via 12V charging cables and activated manually each evening. The air sampling process involved drawing air through pre-labelled, thermal desorption sampling tubes that capture airborne chemicals for later laboratory analysis. Before handling the tubes, operators sanitised their hands and used cotton gloves to avoid contamination. Tubes were labelled with serial numbers and placed into designated sampling trays, allowing traceability of each collected sample. Upon completion of sampling each evening, sample tubes were placed in a cool box with gel ice packs to keep them at a cool temperature for transport back to Jinja Referral Hospital for storage before shipping via airfreight to Loughborough University for analysis by gas chromatography.

Entomological endpoint sample sizes were not powered to detect a significant reduction by the spatial emanator, compared with the control arm due to logistics and resource considerations. These endpoints were conducted for exploratory purposes and to support the interpretation of the epidemiological primary endpoint.

VHTs were trained to actively identify and report adverse events during the 12 months of the trial, and for up to one month afterwards. Participants were advised to report adverse events to VHTs at any time during the trial. Reports were reviewed by the trial clinician for relatedness and severity, and referrals were made to health centres as appropriate. All adverse events were followed up until resolution. Post-hoc adverse-event data were collected during monthly visits to replace the refill coins and at household surveys.

### Outcomes

The main trial objective was to estimate the effect of an active transfluthrin-based spatial emanator on malaria infection prevalence among children aged ≤5 years, as measured by RDT or microscopy. Secondary entomological endpoints were adult female anopheline mosquito composition, population density and human biting rate, and prevalence of resistance to pyrethroids. Additional secondary endpoints are outlined in Clinicaltrials.gov (NCT06232954) with outcomes reported in the protocol. A full trial timeline is shown (Fig 3).

**Fig 3:**
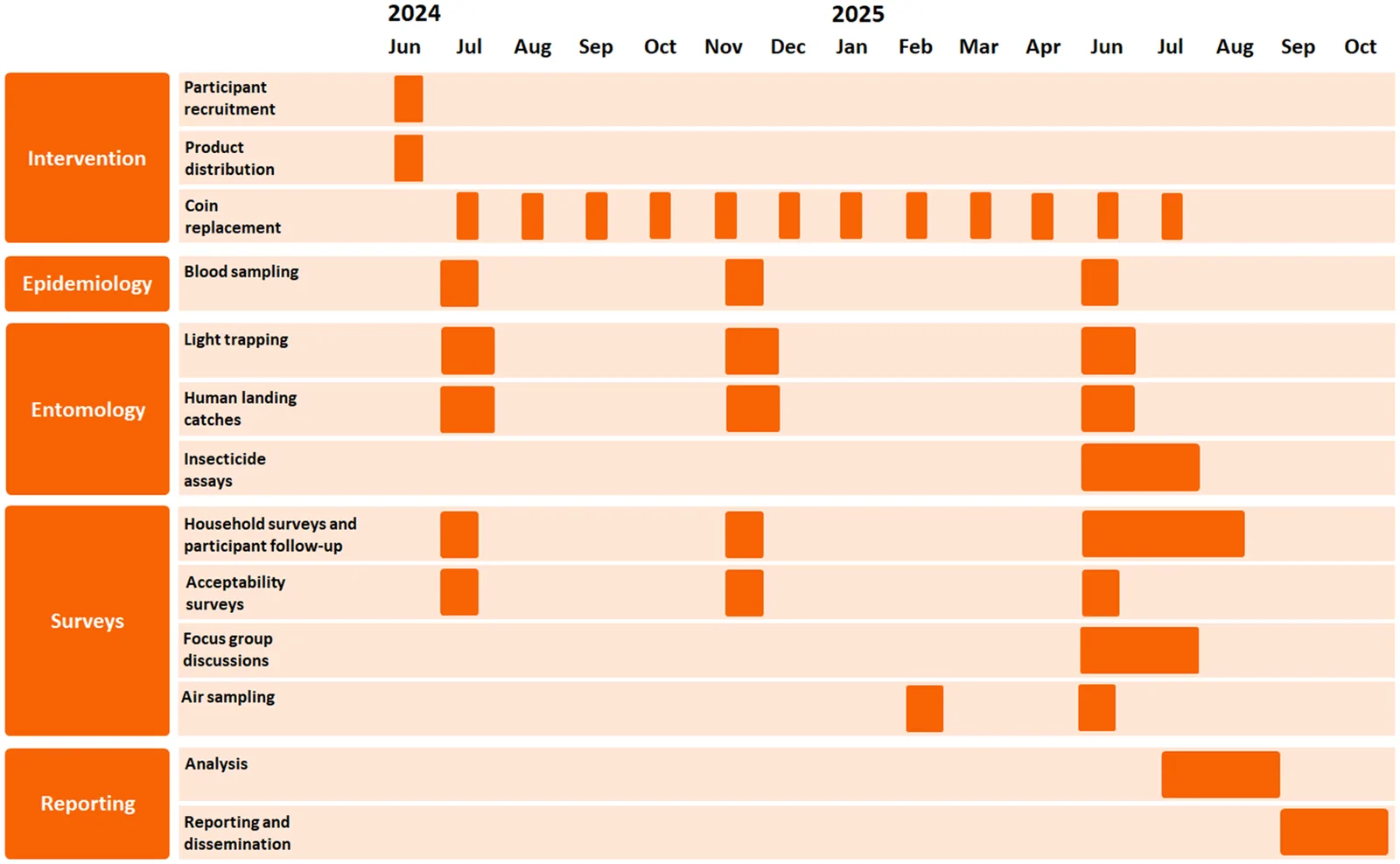
Trial timeline.

### Statistical analysis

The primary endpoint for analysis was malaria infection status among sampled children aged ≤5 years at each survey round. Because children were sampled independently at baseline, 6 months, and 12 months, analyses were conducted as repeated cross-sectional comparisons rather than longitudinal within-participant analyses. Malaria infection status at each follow-up timepoint was compared between intervention and control using mixed-effects logistic regression models with a logit link and a random intercept for study cluster to account for potential confounding factors and the non-independence of observations within clusters. Models were fitted separately for the 6-month and 12-month follow-up timepoints. Unadjusted models included trial arm as the only fixed effect. The primary adjusted models included trial arm and baseline cluster-level malaria prevalence as fixed effects. Baseline cluster-level malaria prevalence was calculated as the mean malaria infection status among sampled children in each cluster at baseline. No formal weighting was applied. Models were fitted using participant level records, with clustering accounted for the random intercept for study cluster. Baseline-adjusted estimated were treated as the primary estimates because baseline malaria prevalence differed between trial arms. Other variables, including socioeconomic status, district, and land type, were examined in exploratory post hoc subgroup or restricted analyses rather than included in the primary adjusted model. For exploratory subgroup analyses, socioeconomic status (SES) was derived using an asset-based index constructed from electricity access, household transport, and durable goods. These variables were converted into binary asset indicators and combined using principal components analysis, with the first principal component used as the SES score. Households were grouped into high and low SES categories based on the distribution of this index. Additional analyses included exploratory post hoc subgroup analyses by socioeconomic status, district, and land type. These variables were not included in the primary adjusted model.

Missing data were handled using an available case approach and no formal imputation was undertaken. Malaria infection status was defined using a composite of RDT and microscopy results. Participants were classified as malaria-positive where either diagnostic test was positive and as negative where both tests were negative. Where only one test result was available classification was based on the available test result. Diagnostic-specific analyses using RDT and microscopy are presented in the Supporting Information and yielded findings consistent with those of the primary composite endpoint Participants with neither RDT nor microscopy data available were excluded from the primary analysis. The composite endpoint was used to capture malaria infection identified by either diagnostic method and was not introduced as a means of addressing missing data. Additional analyses comprised adjusted models for the primary endpoint and exploratory subgroup analyses by socioeconomic status, district, and land type. The subgroup and restricted analyses were conducted post hoc after reviewing the main trial results and should be regarded as exploratory

For entomological outcomes, mosquito species composition was expressed as the total abundance and species-specific proportions of mosquitoes collected per sample, per household, per night. Differences between treatment arms were analysed using logistic regression models, as described for the primary outcome, with cluster-level random effects.

Mosquito density was reported as the total abundance of mosquitoes, stratified by species. For female *Anopheles* mosquitoes, abundances were further categorised by blood-feeding status (unfed, blood-fed, half gravid, or fully gravid). To account for the overdispersion typical of count data, mosquito abundance was analysed using a mixed-effects negative binomial regression model with a log-link function and a random effect for cluster. Where possible, adjusted models were fitted to account baseline variation. For mosquito species-specific analyses with sparse HLC observations or substantial baseline imbalance between study arms, adjusted models were considered unreliable and estimates were classified as non-estimable rather than interpreted.

Quantification of the temporal changes in concentration of transfluthrin present in the air was calculated as nanograms per litre of air sampled and compared for the sampling time points. The results are presented using summary statistics, in a graphical format and the outcomes were compared across treatment arms.

Adverse events were summarized at cluster level as events per 1,000 population. Cluster-level adverse event rates were compared between trial arms using an independent-samples t-test, with 95% confidence intervals calculated for the difference in mean cluster rates.

Two prespecified outcomes have been deferred due to budget constraints: PCR analyses of bloodspots as an independent measure of human malaria infection, and sporozoite rate analyses of a subsample of collected *Anopheles*. The stored bloodspots and mosquito samples remain available. Subject to additional funding, these studies will be completed and the results reported separately.

## Results

### Trial profile

A total of 11,011 households were enrolled in the trial, of which 9,450 (86%) received an intervention or control device, across 56 clusters in June 2024 (Fig 1). No clusters were lost to follow-up or excluded after randomisation; all 56 clusters (28 per arm) were retained at each time point and included in the analysis. Participant sampling was conducted independently at baseline, 6 months, and 12 months, with different households selected at random at each survey round. The trial duration and endpoint sampling was reduced from 18 months to 12 months, due to resource constraints. A total of 10,105 participants ≤ 5 years of age at the time of enrolment underwent malaria sampling across the full length of the study (Table 2). On average 74 participants were sampled per cluster at baseline, followed by an average of 50 and 56 at each following time point. Before the start of the baseline survey, Jinja and Buikwe districts received bed nets in 2022, achieving an estimated coverage of 88% in Jinja and 90% in Buikwe study areas. Cluster-level assigned spatial emanator coverage was fixed at 86% of the total number of households in each cluster, for both the intervention and control arm, equating to a mean of 170 households per cluster. A device troubleshooting team was trained and equipped to repair or replace faulty units within the field. A total of 5,033 faulty cases were reported across the trial. The mean monthly failure rate across the reporting period was approximately 5%, ranging from 1% to 13%. The majority of faulty device units were due to a broken switch which impaired the fan functionality (37%), damage to the solar panel USB (31%) and inability to charge (26%). The troubleshooting records did not capture the duration for which individual devices remained non-functional before repair or replacement; consequently, non-functional device-months could not be reliably estimated. Functional coverage could therefore not be quantified. Device use was also not directly monitored. Consequently, used coverage and overall effective coverage could not be estimated.

**Table 2:** Population characteristics. Note that the broader household survey population and includes individuals of all ages. It is not restricted to children aged ≤5 years included in the malaria prevalence analysis. Denominators for the malaria prevalence analysis are reported separately.

	Baseline		Interim time point		Endline	
**Control**	**Intervention**	**Control**	**Intervention**	**Control**	**Intervention**
Participants	12,597	12,480	6,693	6,640	6,398	6,277
Age, in years at enrollment, mean (SD)	12.6 (15.4)	13 (15.7)	9.35 (12.8)	10.1 (13.4)	9.91 (13.2)	8.84 (12.7)
Sex (f:m)	54:46	54:46	53:47	54:46	54:46	54:46
Households sampled	2,744	2,797	1,656	1,658	1,684	1,689
Household with open eaves	29%	29%	35%	24%	39%	35%
Clusters	28	28	28	28	28	28

### Malaria prevalence

The intervention ran from June 2024 to June 2025. Data were collected from 56 clusters (28 control, 28 intervention) at baseline, 6 months and 12 months. For each household, a single participant was sampled for malaria testing. At baseline, the target was to sample up to 100 children per cluster. We achieved 2,214 participants in control clusters (mean 79.1 per cluster) and 2,158 in intervention clusters (mean 77.1 per cluster). At follow-up, the target was reduced to approximately 60 children per cluster because of funding constraints. At 6 months, 1,411 and 1,368 participants were sampled in control and intervention clusters respectively (mean 50.4 and 48.9 per cluster), and at 12 months, 1,596 and 1,570 (mean 57.0 and 56.1 per cluster)

At baseline, malaria prevalence as determined by RDT or microscopy, was 27.1% in control clusters and 20.7% in the intervention clusters. Because baseline malaria prevalence differed between trial arms, baseline-adjusted estimates were treated as the primary inferential estimates. Malaria prevalence increased from baseline to 6 months in both trial arms. Because participants were sampled independently at each timepoint, these changes describe population-level prevalence at each survey round rather than within-child changes over time. The absolute difference in malaria prevalence between control and intervention clusters at 6 months was 9.0 percentage. Prevalence was 30.7% in control clusters and 21.7% in intervention clusters. The unadjusted odds ratio (OR) for malaria infection was 0.60 (95% CI: 0.30–1.24, *p* = 0.168), and after adjusting for baseline prevalence, the OR was 0.87 (95% CI: 0.61–1.25, *p* = 0.45), indicating no significant effect of the intervention at this interim time point (Fig 4).

**Fig 4:**
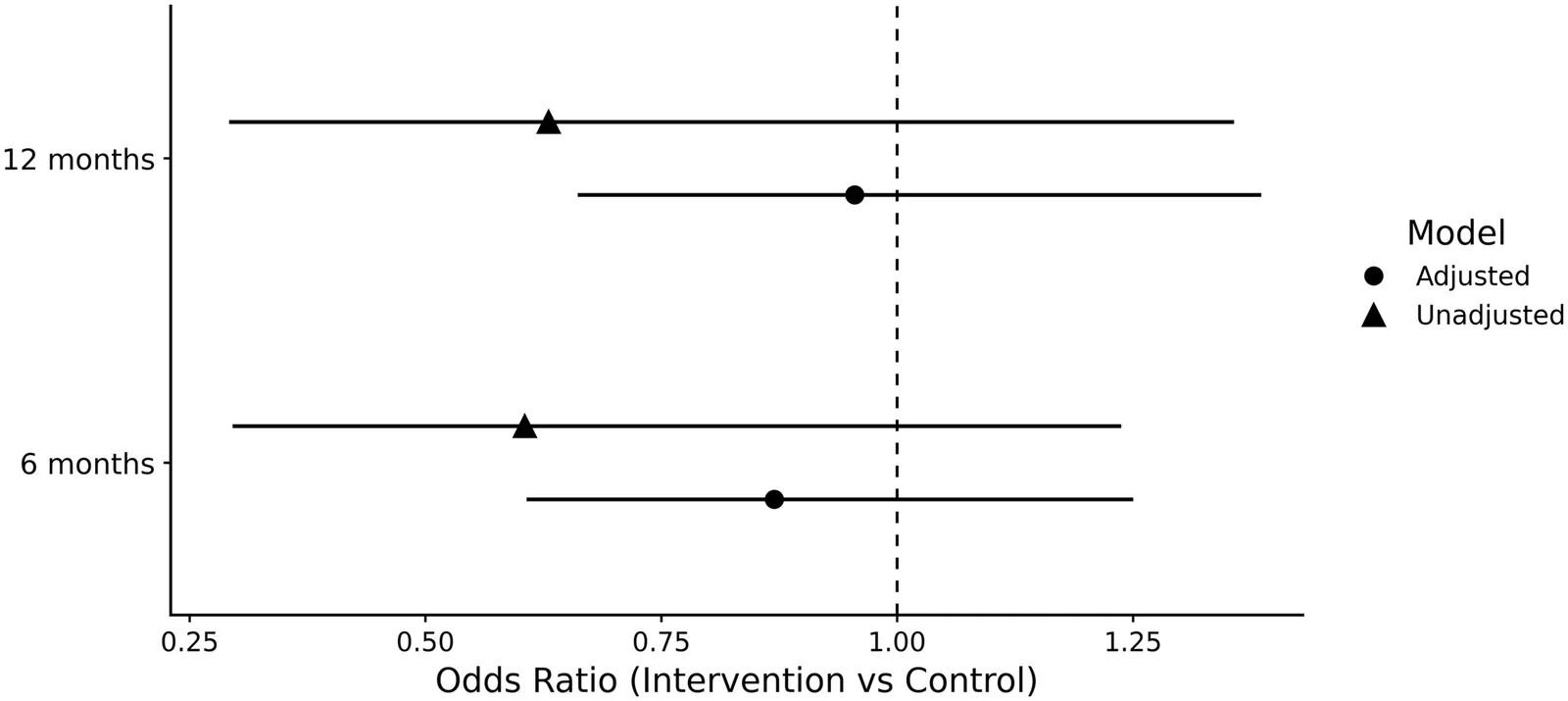
Overall odds ratios for malaria in intervention versus control at 6 and 12 months. Forest plot showing the estimated odds ratios (ORs) and 95% confidence intervals for malaria infection prevalence in intervention versus control clusters at 6 and 12 months, from repeated cross-sectional surveys. Circles represent adjusted models controlling for baseline covariates, while triangles represent unadjusted models. The dashed vertical line indicates an odds ratio of 1.0, corresponding to no difference between intervention and control groups. Values below 1 suggest reduced odds of the outcome in the intervention group relative to the control group.

The absolute difference in malaria prevalence between control and intervention clusters at 12 months was 8.7 percentage points. Prevalence was 41.9% in control clusters and 33.2% in intervention clusters. The unadjusted OR was 0.63 (95% CI: 0.29–1.36, *p* = 0.239), and the adjusted OR was 0.96 (95% CI: 0.66–1.39, *p* = 0.81). Inclusion of baseline prevalence in the adjusted model reduced the apparent effect size at both follow-up time points.

RDT and microscopy-specific prevalence estimates are presented in Supporting Information. RDT-derived prevalence was consistently higher than microscopy-derived prevalence across trial arms and time points, while the composite endpoint produced higher prevalence estimates by capturing infections detected by either diagnostic method. RDT results were unavailable or uninterpretable for 278 malaria-sampling records (2.7%), while microscopy results were available for all records included in the analysis

Among low SES households, prevalence was lower in intervention clusters at 6 and 12 months. However, these difference were also apparent at the study baseline, and the adjusted odds ratios showed no evidence of an intervention effect (6 months: OR 0.77, 95% CI 0.520–1.15; 12 months: OR 0.86, 95% CI 0.56–1.33). A similar pattern was observed among high SES households, where the differences between arms were small and all confidence intervals overlapped to include one (6 months: OR 1.01, 95% CI 0.62–1.63; 12 months: OR 1.04, 95% CI 0.69–1.56).

Examining malaria prevalence in each district separately, we found evidence of differences in outcomes between SES groups. In Jinja, as the study progressed through 6 and 12 months, prevalence declined more substantially in the intervention arm than in the control arm, indicating a trend towards a stronger effect of the intervention in this population (6-month prevalence was 27.9% in intervention arm and 40.3% in control arm, compared to 31.5% and 26.3%, respectively at baseline; adjusted OR = 0.68, 95% CI 0.45–1.03, *p* = 0.07). No significant difference was observed among high-SES Jinja households (adjusted OR = 0.92, 95% CI 0.55–1.54, *p* = 0.745 at 6 months and OR = 95% CI 0.53–1.53, *p* = 0.697 at 12 months). In Buikwe, overall prevalence was lower, and no consistent trend was observed. In low SES households, adjusted ORs were 1.00 (95% CI 0.49–2.06, *p* = 0.995) at 6 months and 0.76 (95% CI 0.44–1.32, *p* = 0.331) at 12 months; in high-SES households ORs were 1.09 (95% CI 0.51–2.31, *p* = 0.823) at 6 months and 1.05 (95% CI 0.53–2.08, *p* = 0.885). Housing characteristics differed between SES groups; enclosed ceilings were present in 17% of low-SES households compared with 6.9% of high-SES households.

### Entomological outcomes

The study design was not powered to detect a significant effect of the intervention in entomological endpoints. Some numerical effects were observed. Mosquito density and species composition were reported from indoor light trapping conducted in seven households per cluster at baseline, 6 months, and 12 months. Across all sampling time points, *Anopheles* spp*.* accounted for 25.3% of total mosquito captures in control clusters and 26.3% in intervention clusters (Table 3). Overall trap catches were lower in the intervention arm (4,209 mosquitoes) compared with the control (8,914). This difference reflects higher baseline mosquito densities in control clusters, combined with seasonal declines observed in both trial arms, rather than evidence of a sustained intervention effect. The predominant species were *Anopheles funestus* and *An. gambiae s.l.,* comprising 62.6% and 37.6% of Anopheles catches in control clusters, and 54.2% and 37.8% in intervention clusters. The composition varied between clusters and districts, with *An. funestus* predominating in Jinja (67.8% of *Anopheles*) and *An. gambiae s.l*. more frequent in Buikwe (83.2% of *Anopheles*).

**Table 3:** Mean density of female Anopheles mosquitoes per trap-night by species, arm, and time point. Densities are from indoor light traps. Incident rate ratios (IRRs) compare intervention to control clusters using mixed-effects negative binomial models. Unadjusted models include treatment arm only; adjusted models control for baseline density and cluster-level variation.

Species	Time point (month)	Control mean	Intervention mean	Unadjusted IRR (95% CI)	P value (unadj.)	Adjusted IRR (95% CI)	p value (adj.)
All *Anopheles* spp.	baseline	4.67	2.26				
	6	3.35	1.24	0.37 (0.14–0.98)	0.045	0.53 (0.22-1.28)	0.158
	12	3.71	2.26	0.61 (0.23–1.62)	0.317	0.92 (0.40–2.12)	0.844
*Anopheles gambiae s.l.*	baseline	1.61	0.63				
	6	1.53	0.76	0.55 (0.22–1.40)	0.211	0.71 (0.28–1.78)	0.462
	12	1.31	0.79	0.52 (0.21–1.32)	0.167	0.59 (0.23–1.49)	0.263
*Anopheles funestus s.l.*	baseline	3.26	1.37				
	6	1.80	0.38	0.12 (0.02–0.64)	0.013	0.27 (0.07–1.02)	0.053
	12	2.34	1.37	0.50 (0.11–2.33)	0.378	1.07 (0.31–3.67)	0.918

Human biting rate (HBR), measured through HLCs, was lower in intervention clusters across all species and time points (Table 4). For all *Anopheles* spp., mean HBR at 6 months was 0.52 landings per person per night in control clusters and just 0.10 in intervention clusters, but this difference was not significant because absolute biting rates were low and variation between clusters was high (unadjusted IRR = 0.26, 95% CI 0.02–3.13, *p* = 0.289). At 12 months, rates remained lower in the intervention arm (0.17 vs 1.36 in the control arm; unadjusted IRR = 0.18, 95% CI 0.03–0.97, *p* = 0.046), though the effect was not significant when adjusted for baseline variation (IRR = 0.36, 95% CI 0.07–1.93, *p* = 0.232). For *An. gambiae s.l*., HBRs were very low at all time points, with mean values of 0.12 and 0.01 at 6 months and 0.59 and 0.08 at 12 months for control and intervention clusters, respectively. No significant differences were detected between arms, and models showed poor convergence due to sparse counts. For *An. funestus s.l*., mean HBR was 0.12 in control and 0.02 in intervention clusters at 6 months (unadjusted IRR = 0.18, 95% CI 0.03–0.99). However, there was substantial baseline imbalance (mean HBR 1.57 in control clusters versus 0.01 in intervention clusters), and the small number of clusters resulted in unstable adjusted models. Adjusted estimates were, therefore, considered non-estimable and are not reported. At 12 months, HBR remained lower in the intervention arm (0.18 vs 0.04), but the difference was not statistically significant.

**Table 4:** Mean human biting rate of mosquitoes per trap-night by species, arm, and time point. HBR was derived from indoor and outdoor human landing catches conducted in ten clusters (five intervention, five control) per time point. Incident rate ratios (IRRs) compare intervention to control clusters using mixed-effects negative binomial models. Unadjusted models include treatment arm only; adjusted models control for baseline density and cluster-level variation. Adjusted IRR not estimable for *Anopheles funestus* due to insufficient sample size and substantial baseline imbalance. The adjusted estimate for *Anopheles gambiae* was retained but should be interpreted cautiously given the sparse data and very wide confidence interval.

Species	Time point (month)	Control mean	Intervention mean	Unadjusted IRR (95% CI)	p value (unadj.)	Adjusted IRR (95% CI)	p value (adj.)
All *Anopheles* spp.	baseline	1.92	0.97				
	6	0.52	0.1	0.26 (0.02–3.13)	0.289	0.51 (0.04–6.20)	0.601
	12	1.36	0.17	0.18 (0.03–0.97)	0.046	0.36 (0.07–1.93)	0.232
*Anopheles gambiae s.l.*	baseline	0.28	0.01				
	6	0.12	0.01	0.55 (0.00–397.96)	0.86	14.81 (0.02–10646.69)	0.422
	12	0.59	0.08	0.21 (0.02–2.71)	0.232	5.63 (0.44–72.62)	0.185
*Anopheles funestus s.l.*	baseline	1.57	0.01				
	6	0.12	0.02	0.18 (0.03–0.99)	0.048	Not presented	
	12	0.18	0.04	0.30 (0.05–1.85)	0.195		

Due to the scarcity of larvae, the susceptibility status to permethrin could only be determined in mosquitoes collected at one site, Iwololo, where the mosquitoes were found to be resistant, with a mortality rate of 74.8%. *Anopheles* collected at two sites in Jinja, Iwololo and Idoome, were exposed to transfluthrin. All were found to be susceptible to transfluthrin, with a mortality rate of 100%. Iwololo was a cluster in the control arm, and Idoome is approximately 5 km from two study clusters, both of which were in the control arm.

### User acceptability

Results of the user acceptance survey and FGDs will be presented in a separate publication.

### Air sampling

Air sampling was conducted in fewer households than planned due to resource constraints. Transfluthrin was undetectable in all sampled control households. In intervention clusters, the majority of samples had concentrations below the level of detection (LOD; 0.03 ng/L). Where detectable, concentrations were found to be higher at 1 h than at 4 h. In intervention clusters, the mean concentration among detected samples was 0.17 ng/L of air sampled at 1 h (maximum 0.62 ng/L) and 0.08 ng/L at 4 h (maximum 0.15 ng/L) (Table 5).

**Table 5:** Trial arm and time point summaries for transfluthrin concentration detected via air samplers. Data are sensitised where lowest observable data are shown as less than 0.03. n = 14 (8 > LOD).

Time point	Trial arm	% records > LOD	Range [ng/L]	Mean (SD) [ng/L]
1 hour	Control	0%	–	–
1 hour	Intervention	36%	0.03–0.62	0.17 (0.22)
4 hours	Control	0%	–	–
4 hours	Intervention	43%	0.03–0.15	0.08 (0.04)

### Adverse events

There were 5,661 adverse events reported in 2,709 participants: 2,805 (49.5%) events in the control arm and 2,856 (50.5%) in the intervention arm (Table 6). We compared cluster-level adverse event rates (events per 1,000 population) between arms using a two-sample t-test on cluster rates; mean (SD) per arm were 739.5 (220.6) in the control arm and 745.0 (195.2), mean difference = 5.51 (95% CI -103.6 to 114.6); p = 0.921. The most commonly recorded adverse events were categorised as respiratory (cough, sneezing; n = 3,025), general (headaches, fever; n = 1,099), and gastrointestinal (diarrhoea, vomiting; n = 794). No serious adverse events reported. In all cases, severity was deemed to be either mild or moderate. At the time of reporting, the majority of events were considered to be possibly related to the spatial emanator (Table 6).

**Table 6:** Summary of adverse events. Significance value calculated using a two-sided z-test for the difference in proportions between intervention and control groups.

Adverse events	Control arm	Intervention arm	Rate difference (95% CI)	P value
	2,805 (49.5%)	2,856 (50.5%)		
Rate per 1000 population (95% CI)	720.2	733.4	13.3	0.488
Severity				
Mild	2,272 (81.0%)	2,224 (77.9%)		
Moderate	533 (19.0%)	632 (22.1%)		
Relatedness				
Not related	878 (31.3%)	909 (31.8%)		
Unlikely related	60 (2.1%)	56 (2.0%)		
Possibly related	1,867 (66.6%)	1,891 (66.2%)		
Category				
Dermatological	95 (3.4%)	98 (3.4%)		
Eye	119 (4.2%)	108 (3.8%)		
Gastrointestinal	412 (14.7%)	382 (13.4%)		
General	520 (18.5%)	579 (20.3%)		
Neurological	155 (5.5%)	168 (5.9%)		
Respiratory	1,504 (53.6%)	1,521 (53.3%)		

## Discussion

Previous work has shown that passive transfluthrin emanators can provide significant protective efficacy against wild pyrethroid-resistant *An. gambiae* vectors [29], and a recent clinical study has provided the first evidence of a reduction in malaria infection risk through use of such tools [7]. Like passive emanators, Mossie-GO was developed to address gaps in malaria control and complement existing vector control interventions. By incorporating a powered fan, it was designed to increase dispersal of the active ingredient and is, therefore, expected to offer enhanced protection compared with devices that lack powered or combustion-based means of increasing volatilisation. The Mossie-GO device demonstrated high levels of protective efficacy in clinical trials under controlled indoor conditions and in experimental huts (Jones et al., in review).

The present study did not demonstrate a lower follow-up malaria infection prevalence in intervention clusters compared with control clusters after adjustment for baseline cluster-level prevalence. Exploratory post hoc subgroup analyses suggested possible heterogeneity by SES and district.For example, in Jinja district, where a reduction in malaria prevalence was observed in the intervention arm, although this did not reach statistical significance. These analyses were not prespecified and should be interpreted as hypothesis-generating only. Whilst we had no prior hypotheses regarding differences in outcomes across districts, low-SES households in Uganda, as elsewhere, may be expected to have higher malaria prevalence [30,31]. There may be multiple factors contributing to this trend, including housing design. Previous studies have observed that houses of individuals of lower SES are more likely to have open structures, with unscreened eaves, gaps in walls, and thatched roofs, which can facilitate mosquito entry and result in higher background biting rates [3]. By contrast, higher socioeconomic position and well-built housing that offers fewer entry points for mosquitoes are associated with lower malaria transmission [32–34]. However, in our trial setting we found that baseline mean malaria prevalence was lower in the low-SES households compared with high SES households in Jinja district. Interestingly, a greater proportion of low-SES households had enclosed ceilings (17%) compared with high-SES households (6.9%), potentially resulting in reduced ventilation and allowing airborne transfluthrin from the emanator to accumulate. Differences in the availability and use of other vector control interventions, as well as sociodemographic factors such as work patterns, time spent indoors versus outdoors and economic motivation to prevent malaria [35], may also have influenced trial adherence and the overall effectiveness of the spatial emanator in these two groups.

Our analysis revealed substantial inter-cluster variability in malaria prevalence. Due to budgetary constraints, we were unable to conduct a survey prior to cluster selection and randomisation that would have allowed us to characterise and account for between-cluster differences at the design stage. Although baseline prevalence data were collected following randomisation, the absence of pre-randomisation data reduced the statistical power to detect differences between arms. When baseline prevalence was included in adjusted models, the apparent effect size was reduced at both follow-up time points, suggesting that a proportion of the observed between-cluster variation was attributable to differences in starting prevalence rather than a true intervention effect. This supports interpreting the baseline-adjusted estimates, rather than crude changes over time or unadjusted between-arm comparisons, as the primary inferential results.

With regards to entomological outcomes, we again found large variation in our baseline data and this reduced the accuracy of modelled outputs. We did not identify a significant reduction in indoor mosquito densities or human biting rate over the 12-month study, although there was an observed decrease in *An. funestus* counts at the interim timepoint in intervention-arm houses. *Anopheles funestus* is highly anthropophilic, with endophilic pre- and post-feeding resting behaviour and indoor, late-night biting patterns [22,36,37]. These characteristics mean that treated nets and indoor residual spraying have typically been effective against this species, where it has been susceptible to the insecticide deployed [38,39], and may explain why we saw the greatest impacts on their number through use of the spatial emanator. Those species that spend less time indoors or opportunistically feed on non-human animals are expected to have had less exposure to the transfluthrin released from the device. In addition, resistance monitoring was limited within the study, and we were unable to fully assess how local patterns of insecticide resistance may have influenced the entomological or epidemiological outcomes; susceptibility cannot be assumed across all clusters and resistance in intervention-arm vectors cannot be ruled out

The airborne concentrations at which transfluthrin affects *Anopheles* have not been fully characterised, and investigations are complicated by the extremely low concentrations at which the compound remains biologically active [40]. In semi-field tests in Vietnam, high mortality of *An. dirus* and *An. minimus* was observed following exposure to airborne transfluthrin, yet most air samples were below the detection limit of the available apparatus (0.32 ng/L), making it difficult to determine thresholds for lethal effects [41]. Sublethal behavioural responses are expected at even lower concentrations, below those required to cause consistent knockdown or mortality. Our own experimental work with Mossie-GO in free-flight chambers found 96% mortality of laboratory-reared *An. gambiae*, at 24 hours post exposure, with estimated concentrations averaging 0.62 ng/L transfluthrin in the air (see Supporting information). The concentrations achieved inside homes, where windows and doors may remain open and where the active ingredient might be absorbed onto walls, soft furnishing and charcoal kept indoors, are likely to be significantly lower. A full exploration of airborne concentrations in participant homes was beyond the resources of the current study but deserves attention in future investigations. We cannot rule out the possibility that the lack of impact identified in the present study were caused by the transfluthrin not being sustained above biologically significant concentrations.

Very few field studies have directly measured airborne transfluthrin concentrations in household conditions once a spatial emanator has been deployed. Our failure to detect transfluthrin in more than 50% of sampled intervention houses may reflect analytical detection constraints, but could alternatively indicate that the device did not consistently emanate sufficient transfluthrin, either through its design or because of sub-optimal participant compliance. We are unable to distinguish between these explanations, either of which could plausibly contribute to the limited epidemiological effect observed. From a safety perspective, transfluthrin is of low acute toxicity. Studies with rats determined a 4-hour median lethal concentration of > 513,000 ng/L air [42], over 800,000 times higher than the concentration achieved in our experimental chambers, suggesting there is no risk to human health through inhalation of the transfluthrin released by the device or by future iterations that release greater doses.

The uncertainty of the effects of the device on entomological outcomes may reflect the mode of action of spatial repellents and the challenges of directly measuring their effects on mosquitoes. The approaches we used for entomological assessment have been widely employed elsewhere in field and semi-field trials for the evaluation of traditional tools as well as for spatial emanators [7,43,44]. While some authors have reported that emanators reduce mosquito entry [45], others have found that indoor light traps underestimate their protective efficacy [46]. Further, HLCs measure only mosquito landing rates and are not sensitive to other effects of vector control tools beyond landing inhibition. They do not directly capture secondary effects such as pre- and post-prandial mortality and may, therefore, underestimate a tool’s impact on malaria transmission potential [47]. Indeed, recent semi-field trials with an alternative transfluthrin-based device found that feeding provides a more robust estimate of protective efficacy than measurements of landing [48].

Overall, the intervention was safe and well tolerated. Most adverse events were mild. Although many events were coded as possibly related to the spatial emanator, the absence of a meaningful between-arm difference provides no evidence of a device-related safety signal, and the observed events are more consistent with background morbidity. The participants of our study reported a positive initial response to the Mossie-GO device, as they have with other spatial emanators [49]. Full analysis of our user acceptance surveys and focus group discussions with participants and village heath teams will be reported separately. However, compliance was not directly monitored throughout the study. Self-reported compliance is known to be unreliable and has been shown to influence the apparent effectiveness of spatial mosquito repellent interventions [50,51]. The demonstrated susceptibility of local vectors to transfluthrin, together with the efficacy observed in controlled evaluations, suggests that the null primary result is more consistent with insufficient exposure to transfluthrin under operational conditions, whether through suboptimal device performance, inconsistent use, or both. The study design cannot distinguish between these explanations.

A further major limitation of our study was the unequal malaria prevalence in control clusters and intervention clusters at baseline, which makes it more difficult to determine whether changes in malaria outcomes were caused by the intervention or by initial differences between groups. Further limitations include incomplete assigned coverage within the trial clusters and potential variability in emanator use through choice or device failure. Device troubleshooting records indicated a mean monthly fault-reporting rate of approximately 5% (range 1–13%), but did not capture the duration of non-functionality before repair or replacement. Therefore, the total actual coverage, based on the time during which households were without a functioning device, could not be quantified. Because the main reported faults affected fan operation and charging, device failures may have reduced effective intervention exposure and attenuated any intervention effect. Although we achieved an average household coverage of 86% at baseline, whether spatial emanators can provide household-level protection or require community-wide adoption has not been fully explored. Modelling of community-wide protection suggests that the effectiveness of spatial emanators could depend on housing densities, ongoing vector control efforts, and other contextual factors [52]. Incomplete coverage within clusters may have diluted the intervention effect by allowing continued transmission from untreated households. It could also have led to diversion of mosquitoes from protected to unprotected households, as has been seen with studies of topical repellents [53]. Further, in addition to the inter-cluster variability mentioned above, baseline malaria prevalence was lower than anticipated and differed from that used in our sample size calculations, reducing the statistical power to detect differences between trial arms. We were unable to expand the number of clusters or households per cluster to compensate. A requirement of powered devices is that users must routinely charge and switch them on, and in areas that are largely off-grid the opportunity to charge mobile phones or use solar power for means other than charging the spatial emanator can be attractive.

To conclude, our large randomized controlled trial did not provide evidence of a significant impact of the transfluthrin-based powered spatial emanator on malaria prevalence in Eastern Uganda. Further studies are needed to better understand the gap between efficacy in controlled environments and effectiveness under operational conditions. Semi-field trials that correlate indoor concentrations of transfluthrin with avoidance behavior, knockdown and mortality of *Anopheles* would be extremely valuable, as would smaller studies with more controlled usage and complete coverage within a village cluster to determine whether human behavior and study compliance were the major limiting factor in our trial. Such limitations may be addressed through behavior change activities or modifications to the device.

## Supporting information

S1 Text Trial protocol. (PDF)

S1 Table Malaria prevalence by diagnostic method, and sub-analyses of malaria prevalence. (PDF)

S2 Text Results of free-flight chamber study. https://doi.org/10.64898/2026.08.13.744622.
(PDF)

S1 Checklist CONSORT checklist. The CONSORT checklist is distributed under the Creative Commons Attribution License (CC BY 4.0). https://doi.org/10.1371/journal.pmed.1004587. Statistical analysis plan is available at LSHTM Data Compass https://doi.org/10.17037/DATA.00005067.
(DOCX)
