# Supplementary material for: Effect of a solar-powered spatial emanator on malaria prevalence in Uganda (Mossie-GO trial): A double-blinded, cluster-randomised, and placebo-controlled trial
Source: PLOS Glob Public Health. 2026 Nov 5;6(11):e0007457. doi: 10.1371/journal.pgph.0007457 (PMC13739182; doi:10.1371/journal.pgph.0007457)
Supplement: Supplementary file 2 [file pgph.0007457.s002.pdf]

# Supplementary files

**S1 Table. Malaria prevalence by diagnostic method and completeness of diagnostic data**

| Timepoint | Trial arm | Participants (n) | RDT prevalence (%) | Microscopy prevalence (%) | Composite prevalence (%) | RDT unavailable (n (%)) | Microscopy unavailable (n (%)) |
|---|---|---|---|---|---|---|---|
| Baseline | Control | 2,214 | 23.3 | 15.5 | 27.1 | 95 (4.3) | 0 (0.0) |
| | Intervention | 2,158 | 17.6 | 11.7 | 20.7 | 68 (3.2) | 0 (0.0) |
| 6 months | Control | 1,411 | 27.6 | 21.6 | 30.7 | 43 (3.0) | 0 (0.0) |
| | Intervention | 1,368 | 19.6 | 14.3 | 21.7 | 32 (2.3) | 0 (0.0) |
| 12 months | Control | 1,596 | 38.4 | 29.8 | 41.9 | 17 (1.1) | 0 (0.0) |
| | Intervention | 1,570 | 29.8 | 22.4 | 33.2 | 23 (1.5) | 0 (0.0) |

# Sub-analyses of malaria prevalence

**Trial districts**

Malaria prevalence differed between the two neighbouring study districts, with a higher prevalence observed in Jinja compared to Buikwe at all timepoints. At 6 months, prevalence was 43.5% in control and 32.0% in intervention clusters in Jinja, compared with 13.9% and 10.7% in Buikwe. At 12 months, prevalence was 57.2% in the control 49.2% in the intervention arm, compared with 21% and 17.4% in Buikwe. These differences were not statistically significant in either district (Jinja: adjusted OR 0.77, 95% CI 0.51–1.17, p 0.219; Buikwe: adjusted OR 1.00, 95% CI 0.55–1.80, p 0.993 at 6 months; and Jinja: 0.88, 95% CI 0.55–1.40, p 0.58; Buikwe: 1.04, 95% CI 0.61–1.77, p 0.881 at 12 months).

**Rural and urban settings**

Little difference was observed between rural and peri-urban locations. In rural clusters, prevalence at 6 months was 36.8% in control and 25.6% in intervention clusters (adjusted OR = 0.86, 95% CI 0.58–1.27, p = 0.442). In urban clusters, prevalence was lower overall and differences between arms were minimal (adjusted OR = 0.90, 95% CI 0.41–1.94, p = 0.78). Similar patterns were observed at 12 months.

**Method of malaria diagnosis**

Malaria was confirmed by either RDT or microscopy. Patterns were consistent across both diagnostic approaches. Using microscopy, prevalence at 6 months was 32.1% in control and 22.3% in intervention clusters (adjusted OR = 0.87, 95% CI 0.61–1.25, $p$ = 0.452). Equivalent results were obtained using RDTs (adjusted OR = 0.87, 95% CI 0.61–1.25, $p$ = 0.459). At 12 months, microscopy and RDT results remained closely aligned, with adjusted ORs of 0.96 (95% CI 0.66–1.39, $p$ = 0.813) and 0.97 (95% CI 0.67–1.40, $p$ = 0.861), respectively.

Reported fever in the preceding two weeks among children under five followed a similar trend, with lower prevalence in intervention clusters but no significant difference. At 6 months, fever prevalence was 40.1% in control and 33.3% in intervention clusters (adjusted OR = 0.90, 95% CI 0.59–1.37, $p$ = 0.619). At 12 months, corresponding values were 39.4% and 34.7% (adjusted OR = 0.96, 95% CI 0.68–1.35, $p$ = 0.799).
